# Novel biomarkers for post-contrast acute kidney injury identified from long non-coding RNA expression profiles

**DOI:** 10.7150/ijbs.45294

**Published:** 2021-02-17

**Authors:** Guanzhong Chen, Bowen Liu, Shiqun Chen, Huanqiang Li, Jin Liu, Ziling Mai, Enzhao Chen, Chunyun Zhou, Guoli Sun, Zhaodong Guo, Li Lei, Shanyi Huang, Liyao Zhang, Min Li, Ning Tan, Hong Li, Yulin Liao, Jia Liu, Jiyan Chen, Yong Liu

**Affiliations:** 1 Department of Cardiology, Guangdong Provincial Key Laboratory of Coronary Heart Disease Prevention, Guangdong Cardiovascular Institute, Guangdong Provincial People's Hospital, Guangdong Academy of Medical Sciences, Guangzhou, 510000, Guangdong, China.; 2 Guangdong Provincial People's Hospital, School of Medicine, South China University of Technology, Guangzhou, 510000, Guangdong, China.; 3 The Second School of Clinical Medicine, Southern Medical University, Guangzhou, 510515, Guangdong, China.; 4 School of Medicine, South China University of Technology, Guangzhou, 510000, Guangdong, China.; 5 State Key Laboratory of Organ Failure Research, Department of Cardiology, Nanfang Hospital, Southern Medical University, 1838, Guangzhou Avenue North, Guangzhou 510515, China.; 6 Guangzhou Jingke Bioscience Center, Guangzhou, 510006, Guangdong, China.

**Keywords:** post-contrast acute kidney injury, long non-coding RNA, biomarker, bioinformatics

## Abstract

**Background:** Post-contrast acute kidney injury (PC-AKI) is a severe complication of cardiac catheterization. Emerging evidence indicated that long non-coding RNAs (lncRNAs) could serve as biomarkers for various diseases. However, the lncRNA expression profile and potential biomarkers in PC-AKI remain unclear. This study aimed to investigate novel lncRNA biomarkers for the early detection of PC-AKI.

**Methods:** lncRNA profile in the kidney tissues of PC-AKI rats was evaluated through RNA sequencing. Potential lncRNA biomarkers were identified through human-rat homology analysis, kidney and blood filtering in rats and verified in 112 clinical samples. The expression patterns of the candidate lncRNAs were detected in HK-2 cells and rat models to evaluate their potential for early detection.

**Results:** In total, 357 lncRNAs were found to be differentially expressed in PC-AKI. We identified lnc-HILPDA and lnc-PRND were conservative and remarkably upregulated in both kidneys and blood from rats and the blood of PC-AKI patients; these lncRNAs can precisely distinguish PC-AKI patients (area under the curve (AUC) values of 0.885 and 0.875, respectively). The combination of these two lncRNAs exhibited improved accuracy for predicting PC-AKI, with 100% sensitivity and 83.93% specificity. Time-course experiments showed that the significant difference was first noted in the blood of PC-AKI rats at 12 h for lnc-HILPDA and 24 h for lnc-PRND.

**Conclusion:** Our study revealed that lnc-HILPDA and lnc-PRND may serve as the novel biomarkers for early detection and profoundly affect the clinical stratification and strategy guidance of PC-AKI.

## Introduction

Post-contrast acute kidney injury (PC-AKI), also called contrast-induced acute kidney injury (CI-AKI), is defined as acute kidney injury that occurs after intravascular administration of iodine-based contrast media 1, 2. PC-AKI, which is the third leading cause of hospital-acquired AKI, occurs in 5% to 35.8% of the 80 million people worldwide who receive an iodinated contrast agent and even up to 50% in high-risk patients 3-5. Besides, after a cardiac catheterization, PC-AKI confers an unfavorable prognosis, resulting in 3-fold and 7-fold increases in short-term and long-term mortality, respectively 6, 7. Therefore, accurate diagnosis, which is dependent on identifying an increase in serum creatinine (SCr), is the first step in PC-AKI intervention 8. However, the insensitivity of and delay in SCr testing and the absence of accurate novel biomarkers render early detection of PC-AKI very challenging 9, 10. Additionally, because the key molecular mechanisms and regulatory networks underlying PC-AKI are still unclear, the effectiveness of classical prevention strategies, such as hydration, is controversial and limited, and specific targeted drugs are lacking 11-13. Therefore, screening potential biomarkers and revealing key aspects of the molecular pathogenesis may provide the cornerstone for managing PC-AKI.

Long non-coding RNAs (lncRNAs), a group of single-stranded, non-coding RNAs containing more than 200 nucleotides and having broad regulatory functions across a wide range of biological processes and human ailments, can serve as novel biomarkers to predict various diseases due to their high specificity and detectability 14-16. Recent studies have identified that MALAT1 can effectively regulate proliferation and inflammatory responses in ischemia/reperfusion-induced AKI, while TapSAKI and RP11-354P17.15-001 can sensitively predict the occurrence of AKI in renal allograft recipients 17-19. However, lncRNA expression profiles in PC-AKI and potential lncRNA biomarkers for its detection are still largely unknown.

In this study, we explored the lncRNA expression profile in rat models. Then we conducted bioinformatics analysis and clinical screening to identify lncRNA biomarkers for the early detection of PC-AKI.

## Results

### Establishment of the PC-AKI rat model

**Figure 1:**
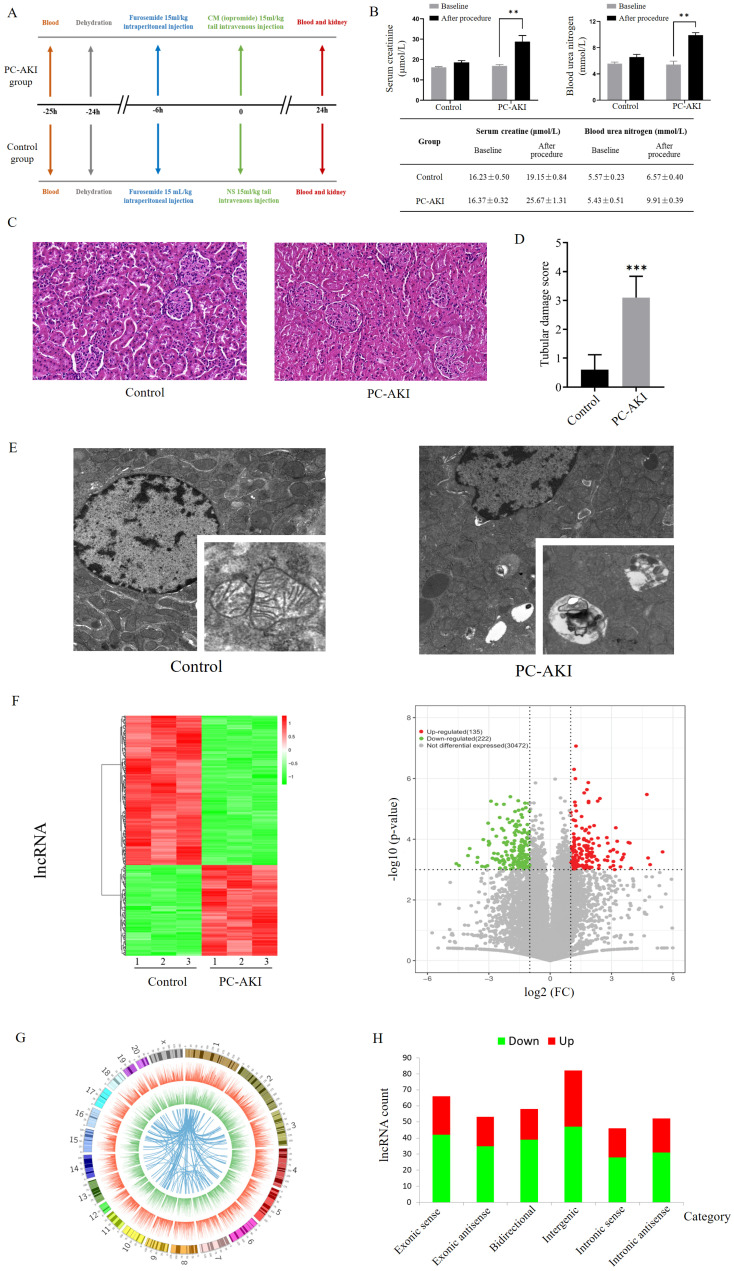
** Establishment and expression profiles of the PC-AKI rat model. (A)** Experimental grouping and operative procedures for establishing the PC-AKI rat model (PC-AKI group) and the control group. **(B)** The concentration of SCr and BUN levels among two groups. **(C)** Representative pathological changes in the kidney tissues of PC-AKI (HE) staining: 40×, scale bar = 50 µm). **(D)** Tubular damage score. Tissue damage was scored according to the percentage of damaged tubules. **(E)** TEM examination of kidney tissues. **(F)** Heat map and volcano plot of differentially expressed lncRNAs in kidney tissues of PC-AKI rat, as determined by RNA sequencing. **(G)** Circos plot showed the chromosomal locations of the differentially expressed mRNAs. The first (outermost) layer of the Circos plot was a chromosomal map of the human genome, and the black and white bars indicated chromosome cytobands. In the second layer (next innermost), the red lines indicated lncRNA expression levels on the chromosomes. In the third layer, the green lines indicated mRNA expression levels. The innermost circle indicated lncRNA-mRNA interaction relationships. **(H)** Types and counts of differentially regulated lncRNAs were classified into six categories according to the genomic loci of their neighboring genes. The measurement data are expressed as the means ± SD. *: P<0.05, **: P<0.01, ***: P<0.001.

As shown in Figure 1A, we randomly divided 6 rats into PC-AKI and control groups. The PC-AKI rat model was established through the classic combination of furosemide and contrast medium (CM). All rats in the PC-AKI group met the PC-AKI diagnostic criteria, SCr and blood urea nitrogen (BUN) concentrations were significantly increased after CM exposure (1.56- and 1.82-fold, respectively, Figure 1B). Haematoxylin-eosin (HE) staining was performed to detect the histopathological changes in PC-AKI. HE staining revealed that the kidney tissues of the control group were basically normal, while those of the PC-AKI group demonstrated typical pathological features of AKI, including severe renal tubular occlusion, extensive tubular cell detachment and infiltration of numerous inflammatory cells. The semiquantitative tubular damage scores in the PC-AKI group (histological score: 3.10 ± 0.73) were significantly increased compared with those in the control group (histological score: 0.60 ± 0.51) (Figures 1C, D). Transmission electron microscope (TEM) examination showed that ultrastructural changes of mitochondria and significant accumulation of lysosomes and autophagosomes were examined in the PC-AKI group (Figures 1E). The elevated SCr concentration, increased kidney injury marker levels, HE staining and TEM examination results indicated the successful establishment of the PC-AKI rat model.

### Overview of lncRNA and mRNA profiles in PC-AKI

To systematically reveal dynamic changes in RNAs in PC-AKI, 6 rat kidney tissues from the PC-AKI and control groups were subjected to next-generation RNA sequencing. A total of 30829 lncRNAs and 11962 mRNAs were detected in PC-AKI tissues. Among these, 135 lncRNAs and 169 mRNAs were significantly upregulated while 222 lncRNAs and 181 mRNAs were significantly downregulated according to the following criteria: |log2 (fold change* vs.* control)| >1 and P<0.001 (Figures 1F, Figure S1A). Analysis of the lncRNA locations showed that the differentially expressed lncRNAs in PC-AKI were widely distributed across all chromosomes except chromosomes 21 and 22. Chromosomes 1 and 2 had the highest number of differentially expressed lncRNAs (Figure 1G). The differentially expressed lncRNAs were classified into the following 6 categories: 18.8% were exonic sense, 14.8% were exonic antisense, 16.2% were bidirectional, 23.0% were intergenic, 12.9% were intronic sense and 14.6% were intronic antisense 20, 21 (Figure 1H).

### Functional analyses and experiments revealed the potential biological processes and pathways mediating PC-AKI

Functional annotation analyses of the differentially expressed genes were performed to predict the roles of RNAs in PC-AKI. Gene ontology (GO) analysis of differentially expressed mRNAs revealed that several cellular processes were enriched, including apoptosis (GO terms: 0097194, execution phase of apoptosis) and autophagy (GO terms: 0006914, autophagy). To validate the apoptosis and autophagy levels in PC-AKI, we conducted terminal deoxynucleotidyl transferase dUTP nick end labeling (TUNEL) assays, Western blotting and TEM in kidney tissues from the rat model (Figure S1B-D, Figure 1E). The results indicated that apoptosis and autophagy might be the potential cell processes to mediate PC-AKI 22, 23. However, further experiments are needed to determine the exact role of these cellular processes in PC-AKI.

Kyoto Encyclopedia of Genes Genomes (KEGG) analysis and Ingenuity pathway analysis (IPA) were conducted to reveal the PI3K/Akt and HIF-1 pathways may mediate the occurrence of PC-AKI (Figure S2A-C). In kidney tissues from the PC-AKI group, HIF-1α was significantly upregulated. As for PI3K/Akt pathway, the ratio p-Akt(S473)/Akt was significantly decreased, while p-Akt(T308)/Akt has no significant changes, which indicates that the renal injury caused by CM was probably due to the suppression effects of the PI3K/Akt pathway via phosphorylation at Ser473 but not at Thr308 24 (Figure S2D). Immunohistochemical staining of kidney tissues further verified that the HIF-1 pathway was activated in PC-AKI (Figure S2E).

### Selection of candidate lncRNAs as potential biomarkers for PC-AKI

**Figure 2:**
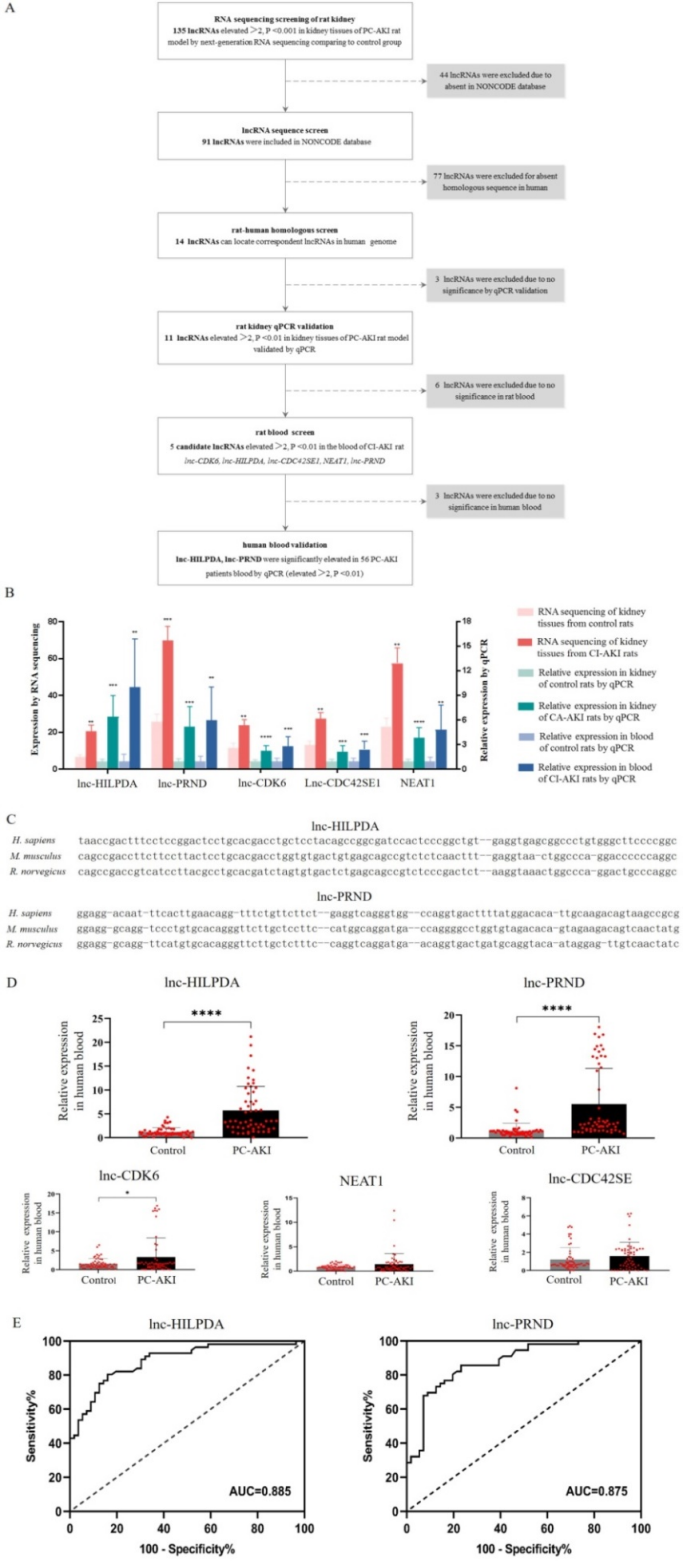
** Selection of candidate lncRNAs and clinical validation of PC-AKI biomarkers. (A)** Flow chart of the candidate lncRNA screening process. **(B)** Expression levels of 5 selected candidate lncRNAs in PC-AKI rat kidney tissues and blood. **(C)** Sequence similarity of lnc-HILPDA and lnc-PRND across species. **(D)** Blood levels of the 5 candidate lncRNAs in 56 PC-AKI patients and 56 matched controls, determined by RT-qPCR. **(E)** The discriminatory potential of lnc-HILPDA and lnc-PRND. Receiver operating characteristic curves were drawn with the fold change data of blood lncRNAs from 56 PC-AKI patients and 56 matched controls. The dashed line indicates the “random guess” diagonal line. AUC indicates the area under the curve. The measurement data are expressed as the means ± SD. *: P<0.05, **: P<0.01, ***: P<0.001, ****: P<0.0001.

Candidate lncRNAs were defined as human-rat homology and significantly upregulated in both kidney tissues and blood samples of PC-AKI rats, and blood samples of human (Figure 2A). First, 135 lncRNAs with greater than 2-fold upregulation and P value <0.001 in PC-AKI rat kidney tissues were selected. Then, 44 lncRNAs were excluded because they were not included in the NONCODE database. Additionally, 77 of these lncRNAs were excluded because of the lack of human-rat homology. The homology was determined by nearby gene anchoring and sequence similarity using the UCSC Genome Browser (http://genome.ucsc.edu/) 25, 26. Some lnc-HILPDA and lnc-PRND sequences conserved across *Homo sapiens*, *Mus musculus* and *Rattus norvegicus*, as determined by the UCSC Genome Browser, are shown in Figure 2C. We validated the expression of the 14 human-rat homologous lncRNAs in PC-AKI rat kidney tissues via qPCR and excluded 3 lncRNAs with an increase <2-fold or a P value >0.01. Then, the expression levels of the remaining 11 lncRNAs were measured in the blood of PC-AKI rats, and 6 were excluded due to an increase of <2-fold or a P value >0.01 in the blood. Ultimately, 5 lncRNAs, namely, lnc-HILPDA, lnc-PRND, lnc-CDK6, lnc-CDC42SE1, and NEAT1, were identified as candidate lncRNA biomarkers for PC-AKI. The expression levels of these 5 candidate lncRNAs in rat kidney tissues and blood are shown in Figure 2B.

### lnc-HILPDA and lnc-PRND as new biomarkers to detect PC-AKI in patients receiving CAG/PCI

To confirm the expression levels of the 5 candidate lncRNAs in patients, 568 consecutive patients receiving coronary angiography (CAG) or percutaneous coronary intervention (PCI) in our center from April 2016 to November 2019 were enrolled. Among the 71 patients who developed PC-AKI, blood samples were available and qualified for 56. We performed a case-control study with these 56 PC-AKI cases and another 56 controls who were matched for age, sex, presence of diabetes mellitus and stage of chronic kidney disease and were randomly selected among patients who did not develop PC-AKI. Clinical characteristics were collected, and no significant differences were observed (Table 1). The expression levels of these 5 candidate lncRNAs in human blood were evaluated via qPCR. After CM exposure, the lnc-HILPDA and lnc-PRND expression levels in blood of PC-AKI patients were significantly higher than those in the controls (mean difference in lncRNA fold changes: lnc-HILPDA, 4.490 [3.135-5.845], P<0.0001; lnc-PRND, 4.334 [2.769-5.899], P<0.0001; Figure 2D). However, the lnc-CDK6, lnc-CDC42SE1, and NEAT1 expression levels did not meet the significant differential expression criteria of fold change >2 and P value <0.01.

Receiver operating characteristic (ROC) analysis was performed to determine the sensitivity and specificity of lnc-HILPDA and lnc-PRND for predicting PC-AKI. As shown in Figure 2E, the levels of these 2 lncRNAs significantly discriminated patients with PC-AKI from those without PC-AKI (lnc-HILPDA: AUC, 0.885 [0.824-0.946]; lnc-PRND: AUC, 0.875 [0.811-0.934]). Based on ROC analysis and the Youden index, the cut-off fold change increase values for lnc-HILPDA and lnc-PRND were established as 1.855 and 1.170, respectively (Table 2). An increase in lnc-HILPDA expression >1.855-fold demonstrated relatively high efficacy for detecting PC-AKI, with a Youden index of 0.64 (sensitivity, 80.36%; specificity, 82.93%). The Youden index was 0.63 when the lnc-PRND cut-off fold change value was 1.170 (sensitivity, 85.71%; specificity, 76.79%). To obtain the optimal diagnostic sensitivity and specificity, we combined these two lncRNAs to predict PC-AKI. This analysis showed that any positive lncRNA combination (lnc-HILPDA increase >1.855-fold or lnc-PRND increase >1.170-fold) yielded the maximized Youden index of 0.84 (sensitivity, 100%; specificity, 83.93%, Table 2). Therefore, lnc-HILPDA and lnc-PRND may be novel biomarkers for detecting PC-AKI in patients receiving CAG/PCI.

### Further evaluation of the biomarker potential of lnc-HILPDA and lnc-PRND

**Figure 3:**
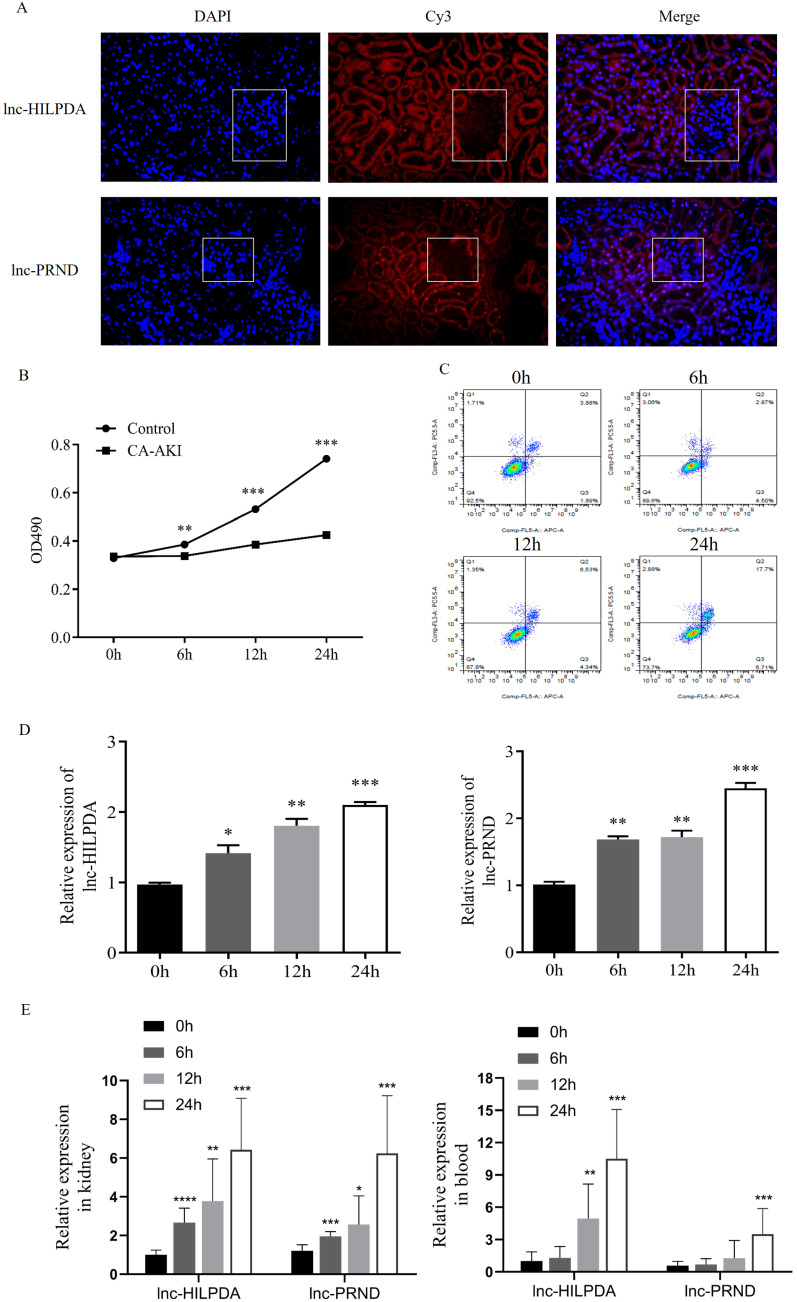
**Localization and expression patterns of lnc-HILPDA and lnc-PRND in PC-AKI. (A)** Localization of lnc-HILPDA and lnc-PRND in kidney tissue. White frames indicate the glomeruli. **(B and C)** Evaluation in the PC-AKI cell models. MTS assays **(B)** and flow cytometry measurement **(C)** of CM-treated HK-2 cells. **(D)** The expression level of lnc-HILPDA and lnc-PRND in CM-treated HK-2 cells over a 24-h time course. **(E)** The expression level of lnc-HILPDA and lnc-PRND in kidney tissues and blood of PC-AKI rats over a 24-h time course. The measurement data are expressed as the means ± SD. *: P<0.05, **: P<0.01, ***: P<0.001.

To investigate the potential of lnc-HILPDA and lnc-PRND as early biomarkers of PC-AKI, we conducted fluorescence *in situ* hybridization (FISH) assays and time-course expression experiments of these two lncRNAs. FISH assays showed that both lnc-HILPDA and lnc-PRND were mainly localized in the tubules (Figure 3A). We treated HK-2 cells with CM to induce cell damage. MTS assays and flow cytometry analysis indicated that HK-2 cells showed damage in a time-dependent manner, with decreased cell viability and an increased apoptotic cell ratio (Figure 3B-C). RT-qPCR was performed on HK-2 cells treated with iopromide at baseline, 6 h, 12 h and 24 h. The temporal trends are shown in Figure 3D. Furthermore, the time-course expression patterns of lnc-HILPDA and lnc-PRND were simultaneously detected in kidney tissues, blood and urine from PC-AKI rat models. The results indicated that both the lnc-HILPDA and lnc-PRND expression levels were significantly different from those in the control group at 6 h in kidney tissues, while a significant difference was first noted in blood at 12 h for lnc-HILPDA and 24 h for lnc-PRND (Figure 3E). However, due to their low abundance in urine, neither lnc-HILPDA nor lnc-PRND could be stably detected in urine (data not shown).

To further evaluate the biomarker potential of lnc-HILPDA and lnc-PRND, we then calculated the correlation of these two lncRNAs with previously validated kidney injury biomarkers, including KIM-1, IL-6, TNF-α, and TIMP2×IGBFP7. As shown in Figure S3A, we found that lnc-HILPDA was positively correlated with TNF-α (r=0.26, P<0.001) and TIMP2×IGBFP7 (r=0.49, P<0.001). Meanwhile, lnc-PRND was positively correlated with TNF-α (r=0.21, P<0.05) and TIMP2×IGBFP7 (r=0.27, P<0.001). The results of the ROC curve analysis for the novel potential biomarkers (KIM-1, IL-6, TNF-α, and TIMP2×IGBFP7) are shown in Figure S3B.

Furthermore, we examined whether lnc-HILPDA and lnc-PRND were specific for distinguishing PC-AKI. An IRI-induced AKI rat model was established by clamping the bilateral kidney pedicles (Figure S4A). The expression levels of both lnc-HILPDA (P=0.69 for kidney; P=0.81 for blood) and lnc-PRND (P=0.67 for kidney; P=0.60 for blood) in the IRI group were similar to those in the PC-AKI group (Figure S4B-C), indicating that these two lncRNAs are not specific for distinguishing PC-AKI from other types of AKI.

### Functional predictions for lnc-HILPDA and lnc-PRND

**Figure 4:**
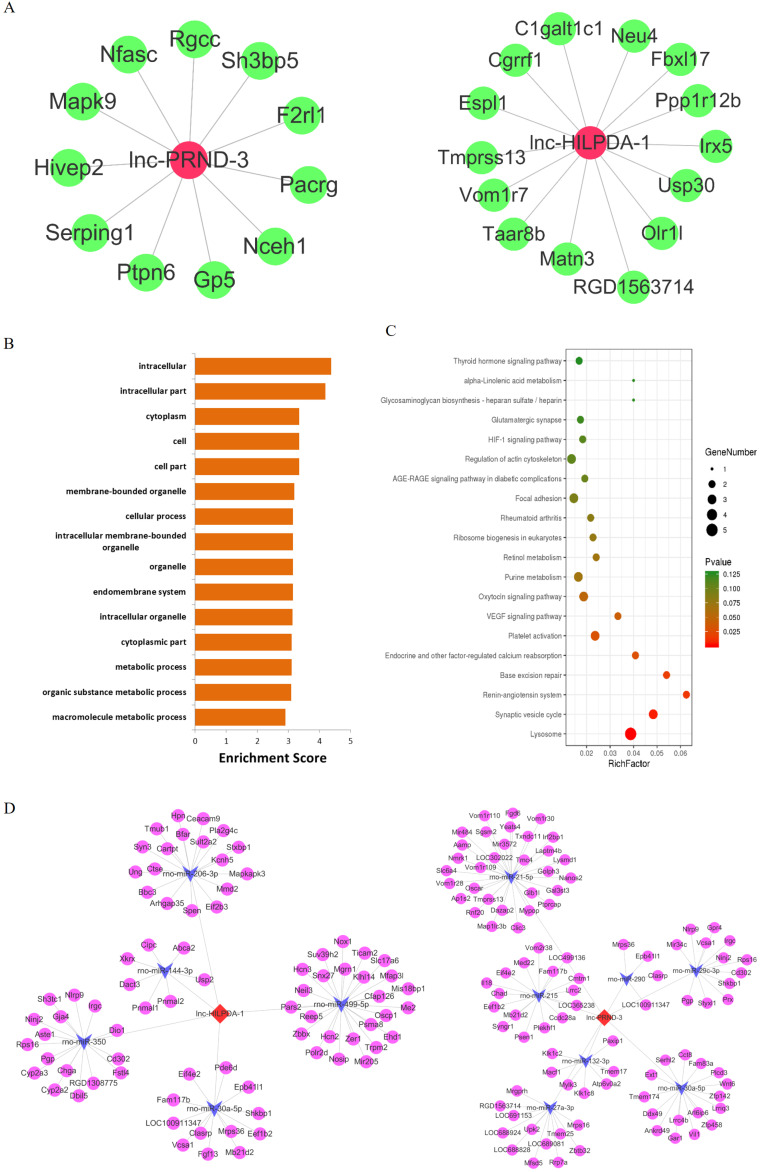
** Functional predictions for lnc-HILPDA and lnc-PRND. (A)** The lncRNA-mRNA coexpression networks of lnc-HILPDA and lnc-PRND. Green nodes, mRNAs; red nodes, lnc-HILPDA or lnc-PRND. **(B)** Biological process annotations with the top 15 enrichment scores determined by GO analysis for lnc-HILPDA- and lnc-PRND-related differentially expressed mRNAs. **(C)** Pathways with the top 20 enrichment scores determined by KEGG pathway analysis for lnc-HILPDA- and lnc-PRND-related differentially expressed mRNAs. **(D)** mRNA-miRNA-lnc-HILPDA and mRNA-miRNA-lnc-PRND ceRNA networks in PC-AKI. Circles, mRNAs; arrows, miRNAs; diamonds, lnc-HILPDA or lnc-PRND.

The considerable biomarker potential of lnc-HILPDA and lnc-PRND indicates that these two lncRNAs may strongly mediate PC-AKI, but their functions in PC-AKI are still unknown. Therefore, focusing on lnc-HILPDA and lnc-PRND, we conducted interaction and functional analyses. We constructed lnc-HILPDA-mRNA and lnc-PRND-mRNA coexpression networks and lnc-HILPDA-miRNA-mRNA and lnc-PRND-miRNA-mRNA competing endogenous RNAs (ceRNA) networks (Figure 4A, D). GO and KEGG pathway analyses of the mRNAs associated with lnc-HLIPDA and lnc-PRND predicted that lnc-HILPDA and lnc-PRND may mediate PC-AKI through the HIF-1 and AGE-RAGE pathways (Figure 4B, C).

## Discussion

In this study, we examined the RNA expression profile of PC-AKI. After rat kidney profiling, human-rat homologous screening and rat blood filtration, we selected 5 candidate lncRNAs for PC-AKI detection. A 112 clinical sample validation demonstrated that lnc-HILPDA and lnc-PRND were significantly increased in PC-AKI patients compared with the controls, which allowed PC-AKI patients to be precisely distinguished, with AUC values of 0.885 and 0.875, respectively. Time-course experiments showed that significant differences of lnc-HILPDA and lnc-PRND were both first noted in the blood of PC-AKI rats at 12 h for lnc-HILPDA and 24 h for lnc-PRND. These observations in this study suggest that lnc-HILPDA and lnc-PRND may serve as the new potential biomarker to distinguish PC-AKI in early phases.

We found that lnc-HILPDA and lnc-PRND may serve as novel biomarkers for PC-AKI, with AUC values of 0.885 and 0.875, respectively. Recent studies have reported that the lncRNAs TapSAKI and RP11-354P17.15-001 can effectively detect the occurrence of AKI 2, 3, which together with our results demonstrates the promising potential of lncRNAs for AKI detection and patient stratification. However, several differences exist between the previous study and ours. First, the populations are different. Previous studies focused on severe AKI patients requiring renal replacement therapy or renal allograft-induced AKI patients. In contrast, our study focused on PC-AKI, a common and specific type of AKI. Second, the screening processes were different. In the previous study, TapSAKI and RP11-354P17.15-001 were directly selected from human blood/urine samples using a two-step screening process, including a small-sample blood/urine lncRNA array analysis and larger sample clinical validation. However, we identified lnc-HILPDA and lnc-PRND through a four-step screening process that included rat kidney RNA sequencing and validation, rat blood screening, human-rat homologous screening and clinical validation. Each of the two different screening processes has advantages and disadvantages. Two-step screening in human samples is convenient and can detect more lncRNAs as candidate biomarkers but cannot identify highly abundant or kidney-specific lncRNAs in the blood and requires more statistical analysis due to the lack of lncRNA expression data in the kidney. Comparatively, four-step screening can narrow down the candidate lncRNAs and select highly abundant kidney biomarkers from combined kidney and blood lncRNA data. However, many data entries may be lost during multiple screening events. Third, prediction efficiencies are different. The previous study showed that TapSAKI and RP11-354P17.15-001 had relatively high prediction efficiencies, with AUC values of 0.80 and 0.76, respectively. In contrast, lnc-HILPD and lnc-PRND, which we identified, showed even better performance, with AUC values of 0.885 and 0.875, respectively. We think the population heterogeneity and the screening criteria may contribute to the differences. Severe AKI may be caused by various factors, such as inflammation, ischemia and medications with renal toxicity 27, 28, and the incidence of renal allograft-induced AKI is affected by many factors, such as donor kidneys, surgeons and immune state 29, 30. In contrast, PC-AKI, which is induced by contrast media, has a similar pathophysiological process 2, 31 and may have less heterogeneity than severe AKI and renal allograft-induced AKI. Additionally, we applied strict screening criteria (fold change>2 and P<0.01) during multiple screening processes. Finally, we identified lnc-PRND and lnc-HILPDA.

As the third leading cause of hospital-acquired AKI 3, PC-AKI was also named CI-AKI 32. The 2018 ESUR guidelines recommended that the term CI-AKI should only be used when a causal relationship can be shown, while PC-AKI is recommended to be used when AKI occurs after intravascular administration of iodine-based contrast media. Although CI-AKI may be better known than PC-AKI, recent studies have suggested that the risk of AKI due to contrast material is overestimated 33. Therefore, in our study, it is better to use “PC-AKI” to indicate the included patients. Previous studies have verified that LNC_000343, LNC_000985 and LNC_000216 may serve as novel biomarkers in a rat PC-AKI model 34. Cheng et al. screened and validated the biomarker potential in a rat model, and their screening process was a relatively simple two-step screening process. In some sense, our four-step screening and human sample validation may have a better chance of identifying potential PC-AKI biomarkers. In this study, we found that lnc-HILPDA and lnc-PRND can distinguish PC-AKI patients with relatively high sensitivity and specificity. Furthermore, the expression of lnc-HILPDA and lnc-PRND in kidney tissues and blood samples was time-dependent, indicating that these two lncRNAs could likely serve as biomarkers in the early stage of PC-AKI. Although showing high sensitivity and specificity, neither lnc-HILPDA nor lnc-PRND could distinguish PC-AKI from other types of AKI, such as IRI-induced AKI. This might be because the pathophysiological mechanisms of PC-AKI are similar to those of IRI-AKI. These two AKI types are both related to oxidative stress 35, vascular endothelial cell damage 36, and imbalanced secretion of vasomotor substances 37, among other factors.

Our study has several limitations. First, the lncRNA profiles were assessed in a rat model rather than in human samples, and the number of rats in each group was limited. As PC-AKI is not a clinical indication for renal biopsy, it would be difficult to acquire PC-AKI patient samples. Although we validated the lncRNA biomarkers in human blood, our profiles and analysis should still be considered when applied to humans. Second, the lncRNA screening process can be further improved. Due to the poor conservation of lncRNAs between humans and rats, the human-rat homologous screening may result in the unavailability of RNA data in humans. Third, the lncRNA biomarker validation was performed on monocentric, small-scale clinical samples, and the efficiency of these biomarkers requires further validation in large-scale, multicentric clinical trials. Moreover, the biological mechanisms by which lnc-HILPDA and lnc-PRND contribute to PC-AKI remain unclear, and further investigations into their functions may provide novel targets and strategies.

In conclusion, this study offers the first analysis of lncRNAs in the blood as potential markers of PC-AKI. We found that lnc-HILPDA and lnc-PRND can effectively predict PC-AKI risk after a cardiac catheterization, therefore increasing the probability that lncRNAs may serve as novel biomarkers for PC-AKI in early phases. Due to the potential clinical application of these lncRNAs, PC-AKI patients may receive precise stratification, early detection and better interventions.

## Materials and methods

### Rat model establishment

Male Sprague-Dawley rats (200-220 g) were purchased from Guangdong Animal Center (Guangzhou, China). For the PC-AKI model, rats were deprived of water from 24 h before the experiment until sacrifice. Six hours before CM exposure, rats were administered 15 mL/kg furosemide via intraperitoneal injection. Rats in the PC-AKI group were injected with iopromide (Ultravist, 300 mg iodine/mL, 330 mOsm/kg H_2_O and 9.5 MPa at 37 °C; Bayer AG, Leverkusen, Germany) at a dose of 15 mL/kg body weight via the tail vein over 5 min. Control group rats received an equal volume of normal saline. PC-AKI rats were sacrificed, and the kidneys were excised after CM exposure for 6, 12, or 24h. Blood samples were collected from the orbital vein and aorta ventralis at baseline and after sacrifice. SCr concentrations were measured, and AKI was defined as a 50% increase in the SCr concentration above baseline after contrast administration 38.

For the ischemia-reperfusion injury model, bilateral kidney pedicles were identified through two small paramedial dorsal incisions and clamped for 45 min. The clamps were then released for reperfusion. The wound was then sutured after good renal reperfusion. Control animals underwent the same surgical procedure, except the renal pedicles were not clamped 39. All rats were sacrificed at 24 h after reperfusion. The AKI definition was the same as that for the PC-AKI group.

All experimental procedures were performed in compliance with the Guide for the Care and Use of Laboratory Animals (National Research Council Publication, 8^th^ Edition, 2011) and in accordance with the Chinese Regulations for the Administration of Affairs Concerning Experimental Animals. The animal experimental protocol was approved by the Guangdong General Hospital Ethics Research Committee (No. 2015253). The model was established at the Department of Experimental Animal Research Centre, Southern Medical University (Guangzhou, China).

### Population

Patients who were scheduled for CAG or PCI in the Cardiology Department of Guangdong Cardiovascular Institute between April 2016 and November 2019 were consecutively enrolled in this study. The inclusion criteria were as follows: (1) provision of a signed informed consent form, (2) age ≥ 18, and (3) treatment with elective CAG or PCI in the Cardiology Department of Guangdong Cardiovascular Institute. The exclusion criteria were as follows: (1) death during the procedure; (2) severe heart failure (cardiogenic shock or NYHA IV); (3) end-stage renal failure or receipt of renal transplant; (4) exposure to CM within 1 week before or 72 h after the procedure; (5) allergy to CM; (6) pregnancy, lactation or the presence of a malignant tumor resulting in a life expectancy of <1 year; (7) use of sodium bicarbonate, non-steroidal anti-inflammatory drugs (NSAIDs), metformin, aminoglycosides, cyclosporine, or cisplatin before or within 48 h after the procedure; or (8) severe valvular disease or planned surgery. Non-ionic, iso-osmolar CM and intravenous hydration were used in all patients. The duration and volume of hydration and the use of N-acetylcysteine were determined at the discretion of the physicians. Peripheral blood (5 mL) was collected 24 h before and 24-30 h after the procedure directly into PAXgene Blood RNA tubes (BD Vacutainer, Franklin Lakes, NJ). SCr was measured at the time of admission and within 72 h after the intervention procedure. PC-AKI was defined as an absolute increase in SCr≥0.3 mg/dL or a relative increase in SCr≥50% over baseline within 72 h 1, 9. The estimated glomerular filtration rate (eGFR) was calculated by the Modification of Diet in Renal Disease formula 40. The Mehran risk score was determined as follows: (1) hypotension: score=5; (2) intra-aortic balloon pump: score=5; (3) congestive heart failure: score=5; (4) age >75 years: score=4; (5) anaemia: score=3; (6) diabetes mellitus: score=3; (7) CM volume: score=1 per 100 cc; and (8) score=2 for 40 mL/min per 1.73 m^2^ ≤ eGFR <60 mL/min per 1.73 m^2^, score=4 for 20 mL/min per 1.73 m^2^ ≤ eGFR <40 mL/min per 1.73 m^2^ or score=6 for eGFR <20 mL/min per 1.73 m^2^ 41. The baseline values for the enrolled patients are shown in Table 1. The study protocol strictly complied with the ethical guidelines of the 1975 Declaration of Helsinki and was approved by the Guangdong Provincial Hospital Medical Ethics Committee (No. 2015253). All patients who participated in this programme were fully informed and signed an informed consent form before enrolment.

### Blood collection and RNA isolation

For the patients' peripheral blood samples and rat blood samples, the PAXgene Blood RNA system was used to stabilize RNA and to store the samples for a relatively long time. Whole blood was collected directly into PAXgene Blood RNA tubes (BD Vacutainer, Franklin Lakes, NJ). Total RNA was isolated within 24 h after sample collection with a PAXgene Blood RNA kit (Qiagen, Hilden, Germany) following the manufacturer's protocol. For rat kidney samples, total RNA was harvested and purified with the standard TRIzol reagent (Invitrogen, Carlsbad, CA) according to the manual. The concentration and purity of total RNA were assessed with a NanoDrop 2000 spectrophotometer (Thermo Fisher Scientific, Wilmington, DE). The quality of RNA samples was checked with an Agilent 2100 Bioanalyzer and RNA 6000 Nano Kit (Agilent, Santa Clara, CA, USA). Samples with an RNA integrity number ≥8 were considered qualified for use. Qualified RNA was stored at -80 °C until further analysis.

### Next-generation sequencing and data processing

The lncRNA, microRNA and mRNA profiles in rat kidney tissues were assessed by next-generation sequencing. rRNA-depleted total RNA was purified from 6 kidney tissues from PC-AKI and control rats using a Ribo-Zero rRNA Removal Kit (Epicentre). Libraries were constructed using an Illumina Gene Expression Sample Preparation Kit and sequenced on an Illumina HiSeq^TM^ 2000 system (Beijing Genomics Institute (BGI)) with 50-60 million 2×100 bp paired raw passing filter reads. The raw reads were filtered to obtain the high-quality reads by removing reads containing an adaptor sequence or reads with a percentage of unknown bases (N) exceeding 10%; low-quality reads contained more than 50% unknown bases and had a Q value of <5. The high-quality reads were mapped to the rat reference genome (IRGSP build 5.0) using SOAPaligner/SOAP2, allowing no more than two mismatches in the alignment. Manipulation of the false discovery rate (FDR) was used to determine the threshold P values in multiple testing and analyses.

### Reverse transcription quantitative real-time polymerase chain reaction (RT-qPCR) analysis

RT-qPCR was used to verify the expression of differentially expressed lncRNAs screened via next-generation sequencing in rat and human blood. Reverse transcription was carried out using total RNA (800 ng from PAX). cDNA was synthesized via reverse transcription using a PrimeScript™ RT Reagent Kit (RR037) with random 6mers and oligo dT primers according to the manufacturer's protocols (Takara, Japan), and reactions were performed in an Eppendorf 5331 PCR instrument (Eppendorf, Hamburg, Germany). Then, quantitative real-time PCR was carried out using TB Green® Premix Ex Taq™ II (Tli RNaseH Plus, RR820) according to the manufacturer's protocols (Takara, Japan). The primers used to amplify lncRNAs in the reactions were synthesized by Generay (Shanghai, China). All RT-qPCR primer sequences are shown in Table S1. qPCR was performed in a CFX Connect Real-Time System (Bio-Rad, USA). The relative expression levels of the lncRNAs were normalized to those of the reference gene GAPDH and calculated using the 2^-▲▲CT^ method. All reactions were repeated in triplicate.

### Haematoxylin-eosin staining

Rat kidney tissues were fixed in 10% neutral buffered formalin (NBF) for a minimum of 24 h and embedded in paraffin, after which 4-mm-thick tissue sections were cut using a microtome. Histopathological evaluation of the tissue sections was conducted with HE staining. Finally, tissue morphology was observed. Tubular damage was scored according to the percentage of damaged tubules 42: 0, no damage; 1, <25% damaged; 2, 25-50% damaged; 3, 50-75% damaged; and 4, >75% damaged.

### TdT-mediated dUTP nick end labeling assay

TUNEL assay for cell apoptosis was used on paraffin sections of kidney tissues from rats and was performed according to the instructions for the In-Situ Cell Death Detection Kit (Roche, Basel, Switzerland). Paraffin sections were cut with a rotary microtome (Leica RM2255, Germany). Sections were washed with xylene two times for 5 min each and then soaked one time in each component of a graded ethanol series. Then, the tissues were treated with Proteinase K working fluid for 15-30 min at 21-37 °C. After two washes with PBS, the TUNEL-converter-POD solution reaction mixture was added and then reacted with 3,3'-diaminobenzidine (DAB) for color development. The cells were subsequently analysed under a fluorescence microscope. Positive cells were counted in 5 randomly selected visual fields at 400× magnification.

### Cell Culture

HK-2 cells were provided by the Stem Cell Bank (Chinese Academy of Sciences, Shanghai, China). HK-2 cells were cultured in low glucose DMEM/F12 medium (Gibco™; Thermo Fisher Scientific, Waltham, MA, USA) containing 10% foetal bovine serum (FBS), 0.1% penicillin and streptomycin at 37°C with 5% CO_2_ and sub-cultured when cells reached 80% confluence. In the contrast medium-treated groups, the cells were treated with 150 mg iodide (mg I)/ml Ultravist Contrast Media Injection (370 mg I/ml; Bayer AG, Leverkusen, Germany) for 0, 6, 12, or 24 h in DMEM/F12 with 10% FBS.

### MTS Assay of Cell Viability

HK-2 cell viability was assessed with a CellTiter96™ AQueous One Solution Cell Proliferation Assay (Promega, Madison, WI, USA) according to the manufacturer's protocol. Cells were cultured in 96-well plates containing a final volume of 100 μl/well. Approximately 20 μl of MTS Reagent was added to each well, and the cells were incubated for 2 h at 37 °C. Cell viability percentages were assessed via spectrophotometry at 490 nm.

### Flow Cytometry Analysis of Apoptosis

Cell apoptosis was examined with an APC-Annexin V and 7-amino-actino-mycin D (7-AAD) staining kit (BD Biosciences, Jersey, USA). Briefly, cells were washed twice with cold PBS and then resuspended in 1× Binding Buffer. After the addition of 5 μl of APC-Annexin V and 5 μl of 7-AAD, the cells were incubated for 15 min at 25°C in the dark. Finally, 400 μL of 1× Binding Buffer was added, and the cells were then analysed via flow cytometry within 1 h. Flow cytometry analysis was performed on a BD Biosciences Accuri C6 flow cytometer (Becton, Dickinson and Company, San Jose, CA, USA) with the 488-nm spectral line of an argon-ion laser.

### Western blot analysis

Rat kidney tissues from the PC-AKI group and control group were homogenized and lysed in ice-cold RIPA lysis buffer (Beyotime, Shanghai, China) supplemented with 1% PMSF using a High Flux Tissue Grinder (Next Advance, New York, USA) for 3 min and then centrifuged for 20 min (16300×g, 4 °C). The supernatant was collected, and the protein concentration was evaluated using an Enhanced BCA Protein Assay Kit (Beyotime, Shanghai, China). Equal amounts of protein (30 μg) were size-fractionated in 15% and 12% SDS-PAGE gels and electrophoretically transferred to PVDF membranes. Membranes were blocked in TBST containing 5% non-fat milk for 2 h at room temperature and were then incubated with primary antibodies overnight at 4 °C. The following antibodies were used: anti-Bax (Proteintech, #50599-2, 1:1000), anti-Bcl-2 (Abcam, ab196495, 1:1000), anti-procaspase-3 (Abcam, ab32150, 1:1000), anti-Cleaved Caspase-3 (Abcam, ab32042, 1:2000), anti-P53 (Proteintech, #10442, 1:1000), anti-p-P53 (Cell Signaling Technology, #9284, 1:1000), anti-LC3 (Sigma-Aldrich, L7543, 1:2000), anti-P62 (Cell Signaling Technology, #5114, 1:1000), anti-HIF-1α (Cell Signaling Technology, #79233, 1:1000), anti-PI3K (Abcam, ab11525, 1:1000), anti-Akt (Cell Signaling Technology, #2920, 1:2000), anti-p-Akt-S473 (Cell Signaling Technology, #4060, 1:1000), and anti-p-Akt-T308 (Cell Signaling Technology, #13038, 1:1000) antibodies. Expression levels were normalized by probing the same blots with an anti-GAPDH antibody (Proteintech, #60009-1, 1:8000).

### Immunohistochemistry

Rat kidney tissue slices were fixed in 10% NBF overnight and then cryoprotected in 30% sucrose solution. For immunohistochemistry, after antigen retrieval in citric acid buffer (pH 6.0), the slices were incubated with primary antibodies specific for HIF-1α (purchased from Abcam (Cambridge, UK)) overnight at 37 °C. Then, the slices were incubated with secondary antibody (1:200, Abcam, UK) at 37 °C for 40 min and washed three times with PBS. DAB (Abcam, UK) was added for color development for 2 min to visualize the antibody-antigen complexes. Immunostaining images were acquired using a microscope.

### Fluorescence *in situ* hybridization

Renal sections from the PC-AKI group and control group were incubated at 65°C for 48 h with a Cy3-labelled probe for lnc-HILPDA and lnc-PRND purchased from GeneRay Biotechnology (Shanghai, China). Then, fluorescence signals were scanned at 260 nm using a Zeiss LSM 780 confocal microscope (Carl Zeiss SAS, Germany).

### Transmission Electron Microscopy

Kidney samples (1 mm thick) from each group were carefully immersed in fixative solution (2.5% glutaraldehyde and 2% paraformaldehyde in cacodylate buffer (0.1 M, pH 7.4)) for 24 h. The samples were washed with PBS three times, fixed in 10% buffered glutaraldehyde and 1% osmic acid, dehydrated, embedded, and finally sliced to a thickness of 50-90 nm. The ultrastructure of autophagosomes was observed under a transmission electron microscope.

### Enzyme-linked immunosorbent assay

We used commercially available ELISA kits (Cusabio Biotech, China) to measure IL-6, TNF-α, TIMP2, IGFBP7 and KIM-1 expression levels in patient serum according to the manufacturer's protocols. Each experiment was performed independently and repeated three times.

### Bioinformatics analysis

Differentially expressed genes were screened using the following criteria: (1) fold change >2 for upregulation or downregulation and (2) P value <0.001. GO analysis was applied to predict the underlying biological processes, molecular functions and cellular components in PC-AKI. The KEGG database was also used to analyse the potential pathways mediating the pathogenic process. The significant GO terms and pathways were identified with Fisher's exact test, and the FDR was utilized to correct the P values. Additionally, differentially expressed genes were loaded into the “core analysis” function included in the IPA software and included genes associated with biological processes, canonical pathways, upstream transcriptional regulators, and gene networks. Based on the significantly different expression levels of ncRNAs and mRNAs between the control group and the PC-AKI group, miRanda (http://www.microrna.org/microrna/home.do) was used to construct a ceRNA network by predicting microRNA binding sequence sites in lncRNAs and mRNAs. The coexpression analysis was based on weighted correlation network analysis 43. Differentially expressed lncRNAs and mRNAs with fold changes ≥2 and P values <0.01 were analysed. A soft threshold value of ≥0.8 was the recommended parameter for the coexpression analysis. K-Core scoring was used to identify core genes in the coexpression networks 44. A higher k-core score indicates a more central location of a transcript within a network.

### Statistical Analysis

Continuous variables are presented as the means ± SD or medians (with 25^th^ and 75^th^ percentiles). Differences between Gaussian distributed variables were determined with Student's t-test. The Mann-Whitney U test or Kruskal-Wallis test was used to determine differences between non-normally distributed variables. Pearson correlation analysis and Spearman correlation analysis were performed to analyse Gaussian and non-normally distributed variables, respectively. Categorical variables are reported as percentages and were analysed using either a chi-square test or Fisher's exact test. Receiver operating characteristic curves were used to establish cut-off increase values for lncRNAs in the PC-AKI predictions. All P values are 2-sided, and P values less than 0.05 were considered statistically significant. All statistical analyses were performed using SPSS 16.0 and R 3.5.0 software.

## Supplementary Material

Supplementary figures and tables.

## Figures and Tables

**Table 1:** Baseline demographic and clinical characteristics of the study and validation groups

Characteristics	Control (n=56)	PC-AKI (n=56)	*P* value
Men, n (%)	50 (89.3%)	50 (89.3%)	0.99
Age (years)	63.0±12.0	65.7±12.0	0.24
SBP (mmHg)	131.9±20.17	133.6±25.0	0.70
DBP (mmHg)	75.1±11.7	74.7±13.2	0.91
CM volume (mL)	120.2±66.3	119.5±57.1	0.95
ACS, n (%)	41 (73.2%)	42 (75.0%)	0.83
Prior MI, n (%)	10 (17.9%)	8 (14.3%)	0.61
Prior PCI, n (%)	21 (37.5%)	22 (39.3%)	0.84
Hypertension, n (%)	35 (62.5%)	34 (60.7%)	0.85
Hypotension, n (%)	1 (1.8%)	0 (0.0%)	0.15
Anaemia, n (%)	14 (25.0%)	15 (26.8%)	0.82
Hyperlipidaemia, n (%)	9 (16.1%)	13 (23.2%)	0.34
CHF, n (%)	4 (7.1%)	8 (14.3%)	0.22
DM, n (%)	26 (46.4%)	21 (37.5%)	0.33
ACEI, n (%)	26 (46.4%)	32 (57.1%)	0.25
Statin, n (%)	54 (96.4%)	53 (94.6%)	1.00
IABP, n (%)	2 (3.6%)	4 (7.1%)	0.40
Baseline SCr (μmol/L)	95.1±41.9	101.6±66.5	0.54
eGFR (mL/min per 1.73 m^2^)	84.5±32.2	87.7±39.9	0.63
eGFR≥90, n (%)	25 (44.6%)	24 (42.9%)	0.85
60≤eGFR<90, n (%)	18 (32.1%)	18 (32.1%)	1.00
30≤eGFR<60, n (%)	13 (23.2%)	14 (25.0%)	0.83
**PC-AKI stage**			
PC-AKI stage 1, n (%)	-	50 (89.3%)	-
PC-AKI stage 2, n (%)	-	6 (10.7%)	-
PC-AKI stage 3, n (%)	-	0 (0.0%)	-
Mehran score	5.4±5.0	5.7±4.7	0.74
Mehran score <6, n (%)	32 (57.1%)	33 (58.9%)	0.83
Mehran score 6-10, n (%)	14 (25.0%)	14 (25.0%)	1.00
Mehran score 11-16, n (%)	9 (16.1%)	6 (10.7%)	0.79
Mehran score >16, n (%)	1 (1.8%)	3 (5.4%)	0.17

SBP, systolic blood pressure; DBP, diastolic blood pressure; CM volume, contrast media volume used in procedure; ACS, acute coronary syndrome; prior MI, prior myocardial infarction; prior PCI, prior receipt of percutaneous coronary intervention; CHF, chronic heart failure; DM, diabetes mellitus; ACEI, angiotensin-converting-enzyme inhibitor; IABP, intra-aortic balloon pump; SCr, serum creatinine; eGFR, estimated glomerular filtration rate; CKD, chronic kidney disease. Stage I is defined as an SCr increase of ≥0.3 mg/dl or ≥1.5 to 1.9 times the baseline level; stage II as an SCr increase of ≥2.0 to 2.9 times the baseline level; and stage III as more than 3 times the baseline level.

**Table 2:** Discrimination potential and cut-off increment values of lnc-HILPDA and lnc-PRND for PC-AKI prediction

lncRNAs	Area under the curve	Cut-off point			
		Fold change	Sensitivity %	Specificity %	Youden index
lnc-HLPDA	0.885 [0.824-0.946]	1.855	80.36	83.93	0.64
lnc-PRND	0.875 [0.811-0.939]	1.170	85.71	76.79	0.63
Any positive			100.00	83.93	0.84
Double positive			66.07	100.00	0.66

Any positive, fold change of any 1 of the 2 lncRNAs above the cut-off point; Double positive, fold change of both lncRNAs above the cut-off point.

## Abbreviations

PC-AKI: Post-contrast acute kidney injury
lncRNA: long non-coding RNA
AUC: area under the curve
CI-AKI: contrast-induced acute kidney injury
CM: contrast medium
SCr: serum creatinine
BUN: blood urea nitrogen
HE: Haematoxylin-eosin
TEM: Transmission electron microscope
GO: Gene ontology
TUNEL: terminal deoxynucleotidyl transferase dUTP nick end labelling
KEGG: Kyoto Encyclopedia of Genes Genomes
IPA: Ingenuity pathway analysis
CAG: coronary angiography
PCI: percutaneous coronary intervention
ROC: Receiver operating characteristic
FISH: fluorescence *in situ* hybridization
ceRNA: competing endogenous RNAs
eGFR: estimated glomerular filtration rate
FDR: false discovery rate
RT-qPCR: reverse transcription quantitative real-time polymerase chain reaction
NBF: neutral buffered formalin
FBS: foetal bovine serum

## References

[1] van der Molen AJ, Reimer P, Dekkers IA, Bongartz G, Bellin MF, Bertolotto M (2018). Post-contrast acute kidney injury - Part 1: Definition, clinical features, incidence, role of contrast medium and risk factors: Recommendations for updated ESUR Contrast Medium Safety Committee guidelines. Eur Radiol.

[2] McCullough PA, Choi JP, Feghali GA, Schussler JM, Stoler RM, Vallabahn RC (2016). Contrast-Induced Acute Kidney Injury. J Am Coll Cardiol.

[3] Mehran R, Dangas GD, Weisbord SD (2019). Contrast-Associated Acute Kidney Injury. Reply. N Engl J Med.

[4] Wilhelm-Leen E, Montez-Rath ME, Chertow G (2017). Estimating the Risk of Radiocontrast-Associated Nephropathy. Journal of the American Society of Nephrology: JASN.

[5] Christiansen C (2005). X-ray contrast media-an overview. Toxicology.

[6] Rihal CS, Textor SC, Grill DE, Berger PB, Ting HH, Best PJ (2002). Incidence and prognostic importance of acute renal failure after percutaneous coronary intervention. Circulation.

[7] Sun G, Chen P, Wang K, Li H, Chen S, Liu J (2019). Contrast-Induced Nephropathy and Long-Term Mortality After Percutaneous Coronary Intervention in Patients With Acute Myocardial Infarction. Angiology.

[8] Lameire N, Biesen WV, Vanholder R (2008). Acute kidney injury. Lancet.

[9] Stacul F, van der Molen AJ, Reimer P, Webb JA, Thomsen HS, Morcos SK (2011). Contrast induced nephropathy: updated ESUR Contrast Media Safety Committee guidelines. Eur Radiol.

[10] Edelstein CL (2008). Biomarkers of acute kidney injury. Adv Chronic Kidney Dis.

[11] Fähling M, Seeliger E, Patzak A, Persson PB (2017). Understanding and preventing contrast-induced acute kidney injury. Nature reviews Nephrology.

[12] Subramaniam RM, Suarez-Cuervo C, Wilson RF, Turban S, Zhang A, Sherrod C (2016). Effectiveness of Prevention Strategies for Contrast-Induced Nephropathy: A Systematic Review and Meta-analysis. Ann Intern Med.

[13] Nijssen EC, Rennenberg RJ, Nelemans PJ, Essers BA, Janssen MM, Vermeeren MA (2017). Prophylactic hydration to protect renal function from intravascular iodinated contrast material in patients at high risk of contrast-induced nephropathy (AMACING): a prospective, randomised, phase 3, controlled, open-label, non-inferiority trial. Lancet.

[14] Quinn JJ, Chang HY (2016). Unique features of long non-coding RNA biogenesis and function. Nature reviews Genetics.

[15] Liu Y, Zhang YM, Ma FB, Pan SR, Liu BZ (2019). Long noncoding RNA HOXA11-AS promotes gastric cancer cell proliferation and invasion via SRSF1 and functions as a biomarker in gastric cancer. World J Gastroenterol.

[16] Wang ZF, Hu R, Pang JM, Zhang GZ, Yan W, Li ZN (2018). Serum long noncoding RNA LRB1 as a potential biomarker for predicting the diagnosis and prognosis of human hepatocellular carcinoma Noncoding RNAs in acute kidney injury. Oncol Lett.

[17] Brandenburger T, Salgado Somoza A, Devaux Y, Lorenzen JM (2018). Noncoding RNAs in acute kidney injury. Kidney Int.

[18] Kölling M, Genschel C, Kaucsar T, Hübner A, Rong S, Schmitt R (2018). Hypoxia-induced long non-coding RNA Malat1 is dispensable for renal ischemia/reperfusion-injury. Sci Rep.

[19] Zhou Q, Huang XR, Yu J, Yu X, Lan HY (2015). Long Noncoding RNA Arid2-IR Is a Novel Therapeutic Target for Renal Inflammation. Molecular therapy: the journal of the American Society of Gene Therapy.

[20] Ransohoff JD, Wei Y, Khavari PA (2018). The functions and unique features of long intergenic non-coding RNA. Nature reviews Molecular cell biology.

[21] Bridges MC, Daulagala AC, Kourtidis A (2021). LNCcation: lncRNA localization and function. The Journal of cell biology.

[22] Kirkin V, McEwan DG, Novak I, Dikic I (2009). A role for ubiquitin in selective autophagy. Mol Cell.

[23] Pankiv S, Clausen TH, Lamark T, Brech A, Bruun JA, Outzen H (2007). p62/SQSTM1 binds directly to Atg8/LC3 to facilitate degradation of ubiquitinated protein aggregates by autophagy. The Journal of biological chemistry.

[24] Hu J, Chen R, Jia P, Fang Y, Liu T, Song N (2017). Augmented O-GlcNAc signaling via glucosamine attenuates oxidative stress and apoptosis following contrast-induced acute kidney injury in rats. Free radical biology & medicine.

[25] Gong C, Li Z, Ramanujan K, Clay I, Zhang Y, Lemire-Brachat S (2015). A long non-coding RNA, LncMyoD, regulates skeletal muscle differentiation by blocking IMP2-mediated mRNA translation. Dev Cell.

[26] Sallam T, Jones M, Thomas BJ, Wu X, Gilliland T, Qian K (2018). Transcriptional regulation of macrophage cholesterol efflux and atherogenesis by a long noncoding RNA. Nature medicine.

[27] Ronco C, Bellomo R, Kellum JA (2019). Acute kidney injury. Lancet.

[28] Singbartl K, Kellum JA (2012). AKI in the ICU: definition, epidemiology, risk stratification, and outcomes. Kidney Int.

[29] Almond PS, Matas A, Gillingham K, Dunn DL, Payne WD, Gores P (1993). Risk factors for chronic rejection in renal allograft recipients. Transplantation.

[30] Nankivell BJ, Borrows RJ, Fung CL, O'Connell PJ, Allen RD, Chapman JR (2004). Natural history, risk factors, and impact of subclinical rejection in kidney transplantation. Transplantation.

[31] Vlachopanos G, Schizas D, Hasemaki N, Georgalis A (2019). Pathophysiology of Contrast-Induced Acute Kidney Injury (CIAKI). Curr Pharm Des.

[32] Rear R, Bell RM, Hausenloy DJ (2016). Contrast-induced nephropathy following angiography and cardiac interventions. Heart.

[33] McDonald JS, McDonald RJ, Carter RE, Katzberg RW, Kallmes DF, Williamson EE (2014). Risk of intravenous contrast material-mediated acute kidney injury: a propensity score-matched study stratified by baseline-estimated glomerular filtration rate. Radiology.

[34] Cheng W, Li XW, Xiao YQ, Duan SB, Hadj Abdallah N, Baulies A (2019). Non-coding RNA-Associated ceRNA Networks in a New Contrast-Induced Acute Kidney Injury Rat Model. Molecular therapy Nucleic acids.

[35] Hadj Abdallah N, Baulies A, Bouhlel A, Bejaoui M, Zaouali MA, Ben Mimouna S (2018). Zinc mitigates renal ischemia-reperfusion injury in rats by modulating oxidative stress, endoplasmic reticulum stress, and autophagy. Journal of cellular physiology.

[36] Eltzschig HK, Eckle T (2011). Ischemia and reperfusion-from mechanism to translation. Nature medicine.

[37] Betz B, Möller-Ehrlich K, Kress T, Kniepert J, Schwedhelm E, Böger RH (2013). Increased symmetrical dimethylarginine in ischemic acute kidney injury as a causative factor of renal L-arginine deficiency. Translational research: the journal of laboratory and clinical medicine.

[38] Sun S, Zhang T, Nie P, Hu L, Yu Y, Cui M (2014). A novel rat model of contrast-induced acute kidney injury. Int J Cardiol.

[39] Liu H, Wang L, Weng X, Chen H, Du Y, Diao C (2019). Inhibition of Brd4 alleviates renal ischemia/reperfusion injury-induced apoptosis and endoplasmic reticulum stress by blocking FoxO4-mediated oxidative stress. Redox biology.

[40] K/DOQI clinical practice guidelines for chronic kidney disease (2002). evaluation, classification, and stratification. Am J Kidney Dis.

[41] Mehran R, Aymong ED, Nikolsky E, Lasic Z, Iakovou I, Fahy M (2004). A simple risk score for prediction of contrast-induced nephropathy after percutaneous coronary intervention: development and initial validation. J Am Coll Cardiol.

[42] Xie Y, Xiao J, Fu C, Zhang Z, Ye Z, Zhang X (2018). Ischemic Preconditioning Promotes Autophagy and Alleviates Renal Ischemia/Reperfusion Injury. Biomed Res Int.

[43] Langfelder P, Horvath S (2008). WGCNA: an R package for weighted correlation network analysis. BMC Bioinformatics.

[44] Ravasz E, Somera AL, Mongru DA, Oltvai ZN, Barabási AL (2002). Hierarchical organization of modularity in metabolic networks. Science.

