# Supplementary material for: Novel biomarkers for post-contrast acute kidney injury identified from long non-coding RNA expression profiles
Source: Int J Biol Sci. 2021 Feb 17;17(3):882–96. doi: 10.7150/ijbs.45294 (PMC7975710; doi:10.7150/ijbs.45294)
Supplement: Supplementary file 1 — Supplementary figures and tables. [file ijbsv17p0882s1.pdf]

Table S1. Primer sequences and RT-qPCR conditions.

Species	Gene symbol	Forward (5'-3')	Reverse (5'-3')	Product size (bp)	Annealing T (°C)
Human	GAPDH	TGAACGGGAAGCTCACTGG	GCTTCACCACCTTCTTGATGTC	120	59
	lnc-HILPDA	TTATGGCTATGAGATAGGTTGATC	GACAGATGTTTAGGAAGTAGGGTT	154	55
	lnc-PRND	TCCTGTCCTTCTGGGTTTG	AGCAGTGGAGGCTTTCTATG	104	55
	lnc-CDK6	GTCTTCAGGGCCAACAATACAG	TTTCTTCAAATTCAGCCAAAGC	119	59
	NEAT1	CGAGGTGCCTTTACTACAT	TGGAACCCAGAAGACAGA	181	50
	lnc-CDC42SE1	TTGTCCAAACACCAGGGAAAGAG	TCATGGTCCGGTCAATCCGTCT	159	63
Rat	GAPDH	GCCCACTAAAGGGCATCCTG	CATTGAGAGCAATGCCAGCC	111	60
	NONRATT027865 (lnc-ICE2)	CAAGACACTAAGGGTGACTACCAGA	TCTTCACAGGGAGGAGTTCAGG	110	60
	NONRATT019677 (lnc-SLC20A1)	AGCCTGTTTCAGCCTCGTTCTT	TGCGTTTGAGCGTCTGTTGTCT	153	62
	NONRATT023687 (lnc-ZDHHC18)	CCTCCAACCTGACAACTAATGAAGACA	GCACAGTATCAGACTGCACAAATCCC	180	65
	NONRATT018635 (lnc-PRND)	CTCCTGAACGAATGTCCGAGTT	TGAGACAAACCAATGAGCGAGA	285	60
	NONRATG002817 (NEAT1)	ACCTTGCCTCTGGGTCATCTGG	TGCTTGCCTTGGTGAACGCTAT	245	65

	NONRATT015555 (lnc-CDC42SE1)	ATTCCGCATCCAAAGACGACAG	GCAGCAATCCTAGAGGCCAGTAAG	130	63
	NONRATG017301 (lnc-CDK6)	GGCAGATGTTGGGACTTGGATT	TCACGCAGCATGTTGTGGGTAT	83	62
	NONRATT029217 (lnc-VEGFA)	GCTGTGGACTTGAGTTGGGAGGAG	CCAGGGATGGGTTTGTCGTGTT	194	65
	NONRATT005144 (lnc-ARL16)	TCCTGTCACTGGTTCCCTGCTT	GCCTGAAACTTCTACCCTTGCTGT	243	63
	NONRATT029337 (LINC01127)	CGACTCTGCGACGTGATTTCCT	CGCCTCCTCTGTTCTTTCTGCT	175	63
	NONRATG012854 (lnc-FRRS1)	TTGGCTCACATTCAAGTGGGCTAT	AAATCTGGCGTGGGACGTTTAG	129	63
	NONRATG010899 (lnc-DND1)	GCTTTCGTGGTGCATTGTGGAT	TCACTATCTCGCTCCGGCATTT	110	64
	NONRATT023136 (lnc-IFNK)	CGTGAATCTGCAACATCGCTGCTC	ATGCCTCCCTCCATCGTCCTCT	175	66
	NONRATG016572 (lnc-HILPDA)	TTTCCCAAGAGGCAACATCAAG	CACCACCATCTCAGAGCCACAG	147	62

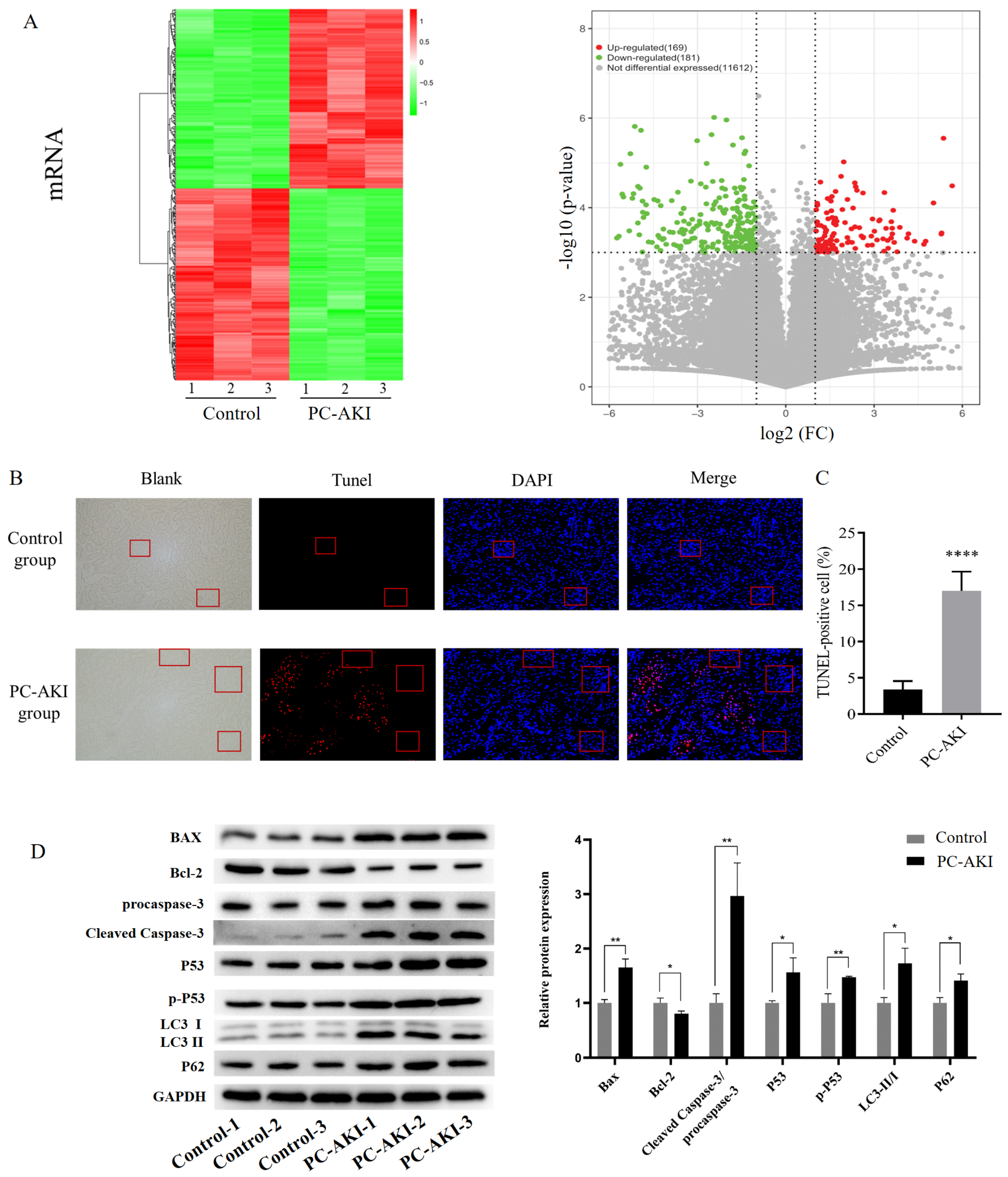

A
mRNA
Control
PC-AKI
Up-regulated(169)
Down-regulated(181)
Not differential expressed(11612)
-log10 (p-value)
log2 (FC)
B
Blank
Tunel
DAPI
Merge
Control group
PC-AKI group
C
TUNEL-positive cell (%)
****
Control
PC-AKI
D
BAX
Bcl-2
procaspase-3
Cleaved Caspase-3
P53
p-P53
LC3 I
LC3 II
P62
GAPDH
Control-1
Control-2
Control-3
PC-AKI-1
PC-AKI-2
PC-AKI-3
Relative protein expression
Control
PC-AKI
Bax
Bcl-2
Cleaved Caspase-3/procaspase-3
P53
p-P53
LC3-II/I
P62

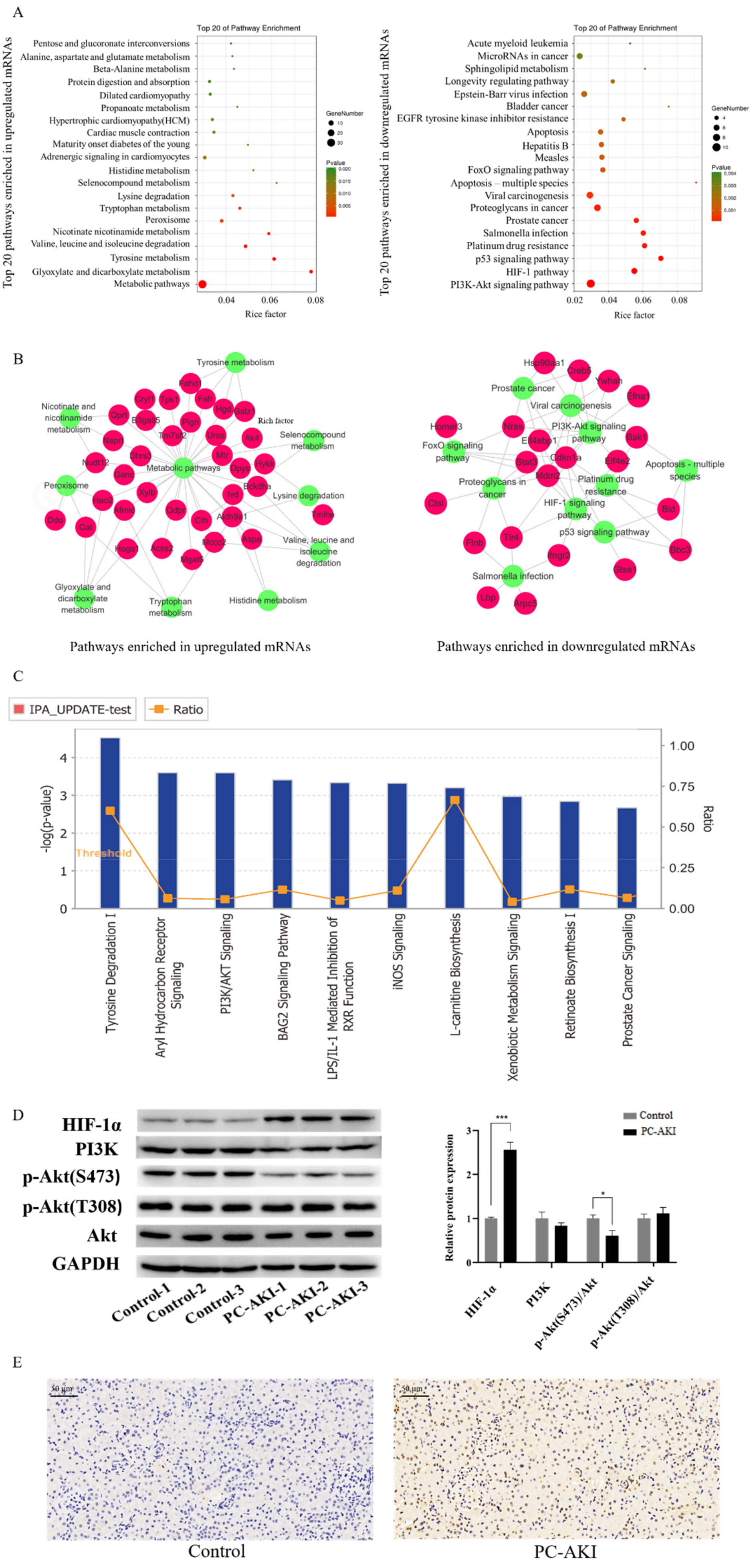
A
Top 20 of Pathway Enrichment
Top 20 pathways enriched in upregulated mRNAs
Pentose and glucoronate interconversions
Alanine, aspartate and glutamate metabolism
Beta-Alanine metabolism
Protein digestion and absorption
Dilated cardiomyopathy
Propanoate metabolism
Hypertrophic cardiomyopathy(HCM)
Cardiac muscle contraction
Maturity onset diabetes of the young
Adrenergic signaling in cardiomyocytes
Histidine metabolism
Selenocompound metabolism
Lysine degradation
Tryptophan metabolism
Peroxisome
Nicotinate nicotinamide metabolism
Valine, leucine and isoleucine degradation
Tyrosine metabolism
Glyoxylate and dicarboxylate metabolism
Metabolic pathways
Rice factor
GeneNumber
Pvalue
Top 20 of Pathway Enrichment
Top 20 pathways enriched in downregulated mRNAs
Acute myeloid leukemia
MicroRNAs in cancer
Sphingolipid metabolism
Longevity regulating pathway
Epstein-Barr virus infection
Bladder cancer
EGFR tyrosine kinase inhibitor resistance
Apoptosis
Hepatitis B
Measles
FoxO signaling pathway
Apoptosis – multiple species
Viral carcinogenesis
Proteoglycans in cancer
Prostate cancer
Salmonella infection
Platinum drug resistance
p53 signaling pathway
HIF-1 pathway
PI3K-Akt signaling pathway
Rice factor
GeneNumber
Pvalue
B
Pathways enriched in upregulated mRNAs
Pathways enriched in downregulated mRNAs
C
IPA_UPDATE-test
Ratio
-log(p-value)
Threshold
Ratio
Tyrosine Degradation I
Aryl Hydrocarbon Receptor Signaling
PI3K/AKT Signaling
BAG2 Signaling Pathway
LPS/IL-1 Mediated Inhibition of RXR Function
iNOS Signaling
L-carnitine Biosynthesis
Xenobiotic Metabolism Signaling
Retinoate Biosynthesis I
Prostate Cancer Signaling
D
HIF-1α
PI3K
p-Akt(S473)
p-Akt(T308)
Akt
GAPDH
Control-1
Control-2
Control-3
PC-AKI-1
PC-AKI-2
PC-AKI-3
Relative protein expression
Control
PC-AKI
HIF-1α
PI3K
p-Akt(S473)/Akt
p-Akt(T308)/Akt
E
50 μm
Control
PC-AKI

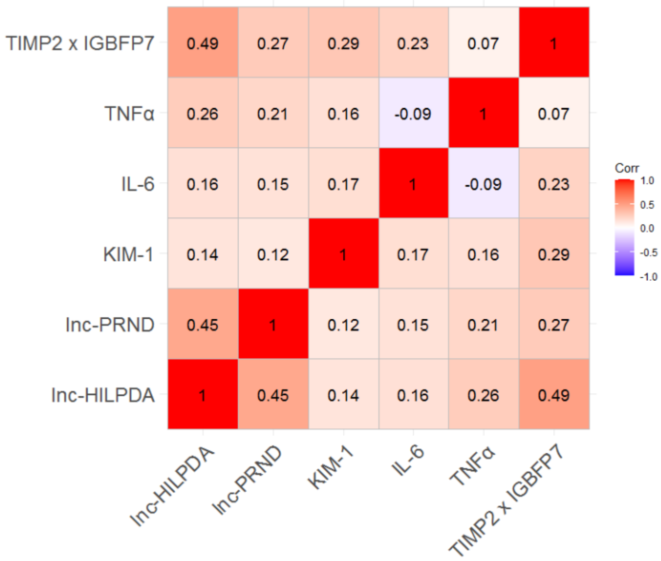
TIMP2 x IGBFP7
TNFα
IL-6
KIM-1
lnc-PRND
lnc-HILPDA
0.49 0.27 0.29 0.23 0.07 1
0.26 0.21 0.16 -0.09 1 0.07
0.16 0.15 0.17 1 -0.09 0.23
0.14 0.12 1 0.17 0.16 0.29
0.45 1 0.12 0.15 0.21 0.27
1 0.45 0.14 0.16 0.26 0.49
Corr
1.0
0.5
0.0
-0.5
-1.0
lnc-HILPDA
lnc-PRND
KIM-1
IL-6
TNFα
TIMP2 x IGBFP7

A

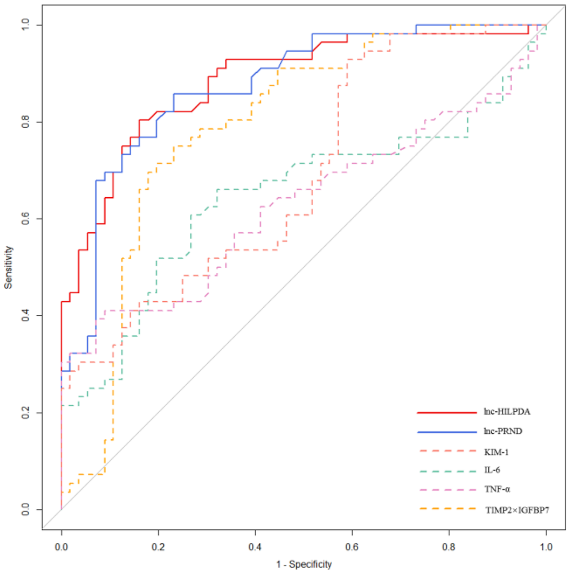
Sensitivity
1 - Specificity
lnc-HILPDA
lnc-PRND
KIM-1
IL-6
TNF-α
TIMP2×IGFBP7

B

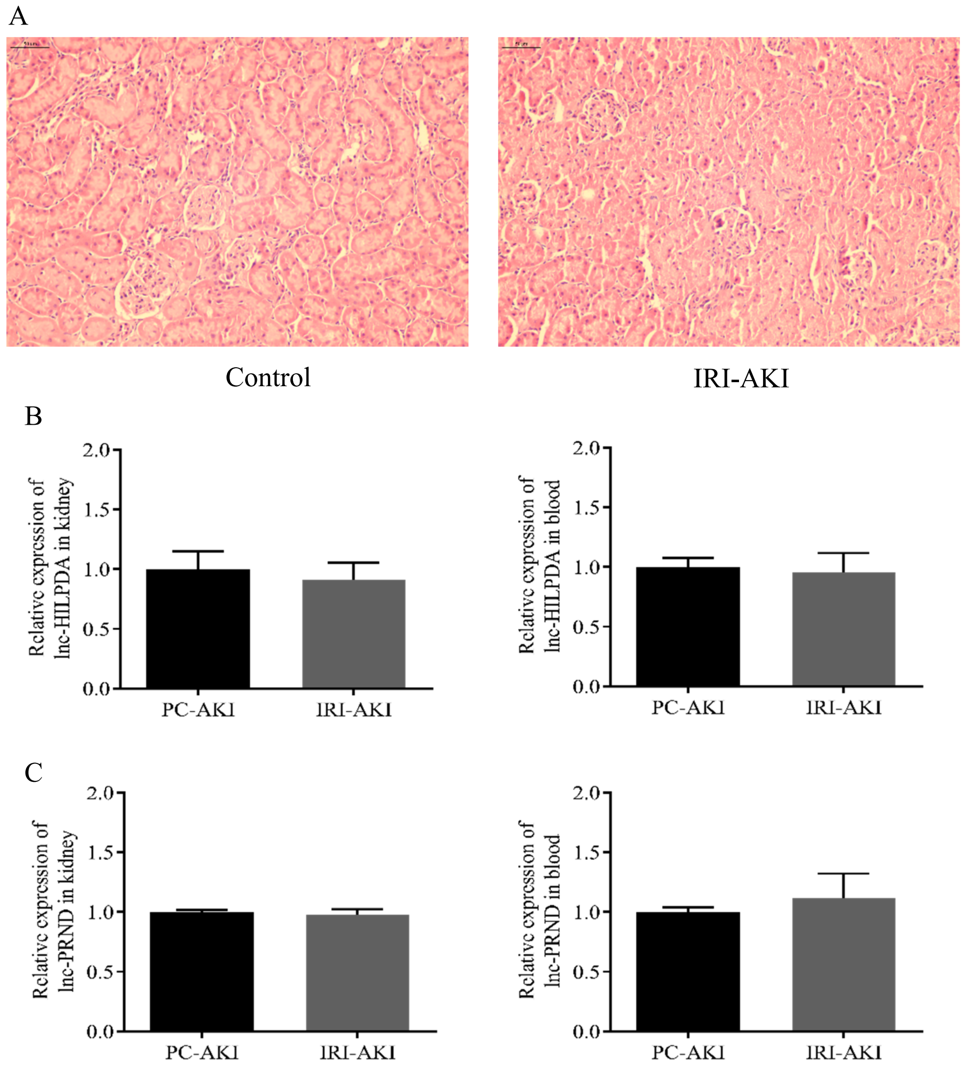

A
Control
IRI-AKI
B
Relative expression of lnc-HILPDA in kidney
2.0
1.5
1.0
0.5
0.0
PC-AKI
IRI-AKI
Relative expression of lnc-HILPDA in blood
C
Relative expression of lnc-PRND in kidney
Relative expression of lnc-PRND in blood

Supplementary Figure Legends

Figure S1 GO analysis and experimental validation of apoptosis and autophagy functions in PC-AKI. (A) Heat map and volcano plot of significantly differentially expressed mRNAs in PC-AKI rat kidney tissues, as determined by RNA sequencing (|log2 (fold change vs. control)| >1 and $P<0.001$). **(B)** TUNEL analysis of kidney tissue from PC-AKI rats and controls. Red frames indicate the glomeruli. **(C)** Quantitative analysis of TUNEL-positive cells. **(D)** Expression of apoptosis and autophagy markers in kidney tissues, as determined by western blot analysis. The measurement data are expressed as the means ± SD. *: $P<0.05$, **: $P<0.01$, ***: $P<0.001$, ****: $P<0.0001$.

Figure S2 Pathway analysis and experimental validation of PI3K/Akt, P53, and HIF-1 pathway enrichment in PC-AKI. (A) Top 20 pathway enrichment scores determined by KEGG pathway analysis of upregulated and downregulated mRNAs. **(B)** Pathway interaction networks in PC-AKI. The green nodes indicate the pathways predicted by KEGG pathway analysis. The red nodes indicate the shared differentially expressed genes connecting different pathways. **(C)** Top 10 pathway determined by IPA analysis of differentially expressed mRNAs. **(D)** Expression of markers in the PI3K/Akt and HIF-1 pathways in kidney tissues evaluated by western blot analysis. The measurement data are expressed as the means ± SD. **(E)** Immunohistochemical analysis of HIF-1 in kidney tissues. *: $P<0.05$, **: $P<0.01$, ***: $P<0.001$.

Figure S3 Correlations analysis between two lnc-RNAs and other validated biomarkers. (A) Spearman's correlation analysis between lnc-HILPDA, lnc-PRND and the other previous validated AKI biomarkers. Red indicates positive correlations while blue indicates negative. The numerical value represents the correlation coefficients. **(B)** ROC curve of lnc-HILPDA, lnc-PRND and the other biomarkers for discrimination between PC-AKI and non-AKI group.

Figure S4 Evaluation of the diagnosis value of lnc-HILPDA and lnc-PRND to distinguish PC-AKI with other kinds of AKI. (A) Representative pathological changes during IRI-AKI in rat kidney tissues (haematoxylin-eosin (HE) staining: 40×, scale bar =50 μm). **(B and C)** Expression level of lnc-HILPDA and lnc-PRND in kidney tissue **(B)** and blood **(C)** of PC-AKI rats and IRI-AKI rats.
